# Nutrigenomics and microbiome shaping the future of personalized medicine: a review article

**DOI:** 10.1186/s43141-023-00599-2

**Published:** 2023-11-22

**Authors:** Neemat M. Kassem, Yassmin A. Abdelmegid, Mahmoud K. El-Sayed, Rana S. Sayed, Mahmoud H. Abdel-Aalla, Hebatallah A. Kassem

**Affiliations:** 1 Clinical and Chemical Pathology Department, Kasr Al Ainy Centre of Clinical Oncology & Nuclear Medicine, School of Medicine, Cairo University, Cairo, Egypt; 2 OBG Department, Kasr Al-Ainy School of Medicine, Cairo University, Cairo, Egypt; 3 Faculty of Medicine, Kasr Al-Ainy School of Medicine, Cairo University, Cairo, Egypt

**Keywords:** Nutrigenomics, Nutrigenetics, Microbiome, MTHFR, Cancer, Personalized Nutrition, Viral infection

## Abstract

The relationship between nutrition and genes has long been hinted at and sometimes plainly associated with certain diseases. Now, after many years of research and coincidental findings, it is believed that this relationship, termed “Nutrigenomics,” is certainly a factor of major importance in various conditions. In this review article, we discuss nutrigenomics, starting with basics definitions and enzymatic functions and ending with its palpable association with cancer. Now, diet is basically what we eat on a daily basis. Everything that enters through our alimentary tract ends up broken down to minute molecules and amino acids. These molecules interact with our microbiome and genome in discreet ways. For instance, we demonstrate how proper intake of probiotics enhances beneficial bacteria and may alleviate IBS and prevent colorectal cancer on the long term. We also show how a diet rich in folic acid is essential for methylenetetrahydrofolate reductase (MTHFR) function, which lowers risk of colorectal cancer. Also, we discuss how certain diets were associated with development of certain cancers. For example, red and processed meat are highly associated with colorectal and prostate cancer, salty diets with stomach cancer, and obesity with breast cancer. The modification of these diets significantly lowered the risk and improved prognosis of these cancers among many others. We also examined how micronutrients had a role in cancer prevention, as vitamin A and C exert anti-carcinogenic effects through their function as antioxidants. In addition, we show how folic acid prevent DNA mutations by enhancing protein methylation processes. Finally, after a systematic review of myriad articles on the etiology and prevention of cancer, we think that diet should be a crucial feature in cancer prevention and treatment programs. In the future, healthy diets and micronutrients may even be able to successively alter the liability to genetic mutations that result in cancer. It also will play a role in boosting treatment and improving prognosis of diagnosed cancers.

*“Let food be thy medicine and medicine be thy food.” ~Hippocrates*

## Background

Nutrigenomics, a relatively new science, is basically the study of the relationship between nutrition and gene expression. Many recent studies showed the significance of nutrition and how it molecularly affects gene expression, postulating that you really *are* what you eat. Nutrition has become a very popular and vital component in determining risk assessment for many diseases plaguing humanity in the current time frame. Plenty of the body’s physiologic or metabolic characteristics are defined by dietary patterns. A simple example is the wide-spread phenomenon of obesity [1]. Additionally, studies showed that the interaction between diet and diseases differs according to genetic and ethnic variations among individuals. For instance, individuals with a C → T substitution in the gene for methylene-tetrahydrofolate reductase (MTHFR) might require more folate than those with the wild-type allele [2]. Another intriguing example of the complex interactions between genetics, diet, and disease is from a study on the incidence of hepatocellular carcinoma in Sudanese population. The study reported that a stronger relationship existed between the risk of developing the disease and the consumption of peanut butter contaminated with aflatoxins in Sudanese people with the glutathione S-transferase M1 null genotype compared to those lacking this genotype [3, 4]. In this review, we discuss the relationship between nutrigenomics and disease. We examine the effect of many food compounds, whether micro or macro, on the development, prognosis, and treatment of cancer and other diseases. We also discuss the association of various dietary factors with different types of cancer. Finally, how nutrition may be used in the prevention of cancer in the future. Slightly controversial, nutrigenomics might seem to be another popular science topic, barely holding a stance as strong as chemotherapeutic or pharmaceutical research in the treatment of diseases. However, in this review, by exploring what studies have shown through the last decade, we are going to show why it should be a vital element in the wholistic approach toward diseases in the future.

### Nutrigenomics

Nutrigenomics is a new, rising field of science that creates a spider web connecting both nutrition and genome activity. According to the field of nutrigenomics, a whole different system of signaling exists in the body predisposing to gene expression; nutrients ingested form the stimuli or the “dietary signals” that are captured by the sensory systems in the cells and directly impacting patterns of gene, protein and metabolite expressions [5]. Nutrigenomics also makes a wide attempt at showcasing the influence of nutrition on the body’s homeostasis; similarly, it aims at identifying certain cellular level interactions that fuel the inflammatory stress pathways to better the understanding of diet related diseases. The field of nutrigenomics clarifies the interaction between bioactive compounds from different food sources and genes. In addition, utilizing the usage of nutritional systems biology to discover and detect biomarkers; the “stress signatures” that predispose to diet related diseases [6].

### Nutrigenetics

**Fig. 1:**
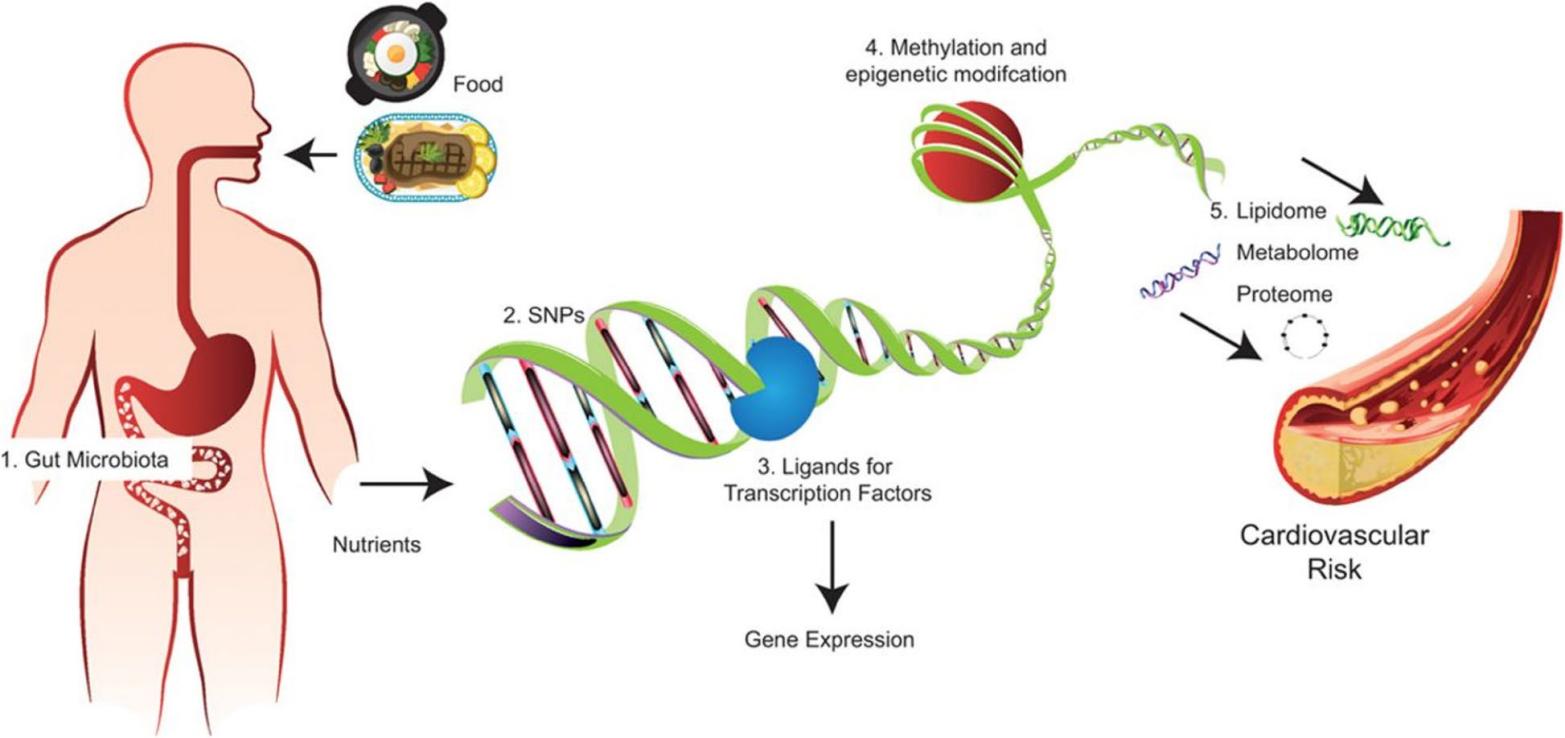
Effect of some foods on the cardiovascular system (CVS) diseases [4]

Nutrogenetics is the relationship between nutrition and the human genome. This relationship defines the gene expression and metabolic response of each individual, influencing health condition and susceptibility to disease (Fig. 1). A fundamental aspect of the genetic approach to disease is an appreciation of human variation. Some of the earliest studies of human biochemical genetics showed considerable variability within and between populations, which is highly relevant for nutrition [7]. Variation in nutritional requirements and interaction of certain nutrients with genetically determined biochemical and metabolic factors suggest different requirements for individuals and call for personalized regimens. This variation, like sex differences, is inborn. Research is defining the mechanisms by which genes influence nutrient absorption, metabolism, and excretion and the mechanisms by which nutrients influence gene expression [8].

### Microbiome

Another important interaction is that between nutrigenomics and the microbiome. The microbiome by definition is the collective genetic material of all the microbes that live in and on the human body. As complicated as it is, the human alimentary tract offers multiple instances where gut microbiome was related to the general health. Moreover, there has been a surge in interest during the past decade in the contribution of the human microbiome to cancer pathogenesis and treatment. Humans carry about 2 kg of microbes, ranging between 500 to 1000 species, and the average adult digests about 500 kg of food annually [9]. The human microbiome comprises the ecosystem of approximately 40 trillion microorganisms, including bacteria, viruses, yeast, and fungi, that colonize our bodies and influence multiple processes, including metabolism, hematopoiesis, and immune function. Though relatively stable throughout adult life, the microbiome has been shown to be altered as a result of infection, antibiotic exposure, lifestyle (e.g., exercise), surgical procedures, diet, and various disease states, including cancer. Research shows that at certain levels, some microbiota may confer benefits to the host, while others may be associated with clinical pathologies. We can take for example atherosclerotic metabolic syndrome. DNA of oral microbiota Veillonella and Streptococcus was found in atherosclerotic plaques of individuals who had these microbes with abundance in their digestive system [10]. On the other hand, microbiota can also protect from atherosclerosis, as it was established in a recent study that showed that *Akkermansia municiphila* reversed Western diet-induced atherosclerosis in mice [11]. In addition, altered balance between the Bacteroidetes and the Firmicutes, two enteric bacterial families, has been associated with obesity state in recent studies. On the other side, we have the well-known probiotics. Probiotics are the only live bioactive compounds recognized by Food and Drug Administration (FDA) as safe. They are combination of live beneficial bacteria and/or yeasts (most commonly *Lactobacillus*, *Bifidobacterium*, and *Saccharomyces*) that naturally live in the body. Due to their inability to colonize the host, they need to be taken regularly in diet as in yogurt or daily supplements. Probiotics confer benefits mostly for lactose intolerance, irritable bowel syndrome, reduction of colorectal cancer risk, and gastric ulcers among other illness. It is also claimed that they have a role in reducing the risks of osteoporosis, obesity, and possibly type 2 diabetes. Another popular example is the fecal replacement therapy, which depends on the replacement of gastrointestinal microbiota in diseased individuals with the fecal material from healthy people. This fecal population can be attenuated to a small number of species creating a microbial product that can improve certain conditions, eventually creating a microbial therapeutic product [12]. This emerging field of gut microbiota and their connection to various diseases is promising as it may offer therapeutic possibilities in the future. Recent studies in this area also take into account nutritional epidemiology across different ethnic groups, differences in macro and micro nutrient intake during lifespan, and personalized tailor-made and/or algorithm-based diets targeting individual nutritional requirements for disease prevention.

### Diet-gene interaction

Metabolizing enzymes are one of the most important factors in activation of carcinogens. Variable individualized responses to toxins were clearly noted in different individuals. Other nutritional factors or lifestyle habits such as obesity, diabetes mellitus (DM), and fasting have been proved to greatly affect the baseline of activity of oxidative enzymes in living organisms. The metabolizing enzymes are fluidly pleomorphic bending to the potential influence of certain genetic alterations that result from our diet-gene interactions. This topic has become a great source for further extensive research expenditures, and here we will discuss few major examples.

- Cytochrome P450 enzymes and genes

Cytochrome P450 (CYP) enzymes are a group of enzymes encoded by P450 genes and expressed as membrane bound proteins mostly found in the endoplasmic reticulum of the liver [13]. CYP enzymes function as monoxygenases and affect oxidation by transfer of one oxygen atom through a number of steps.

For example, the CYP1A2 gene can be induced by indole-3-carbinol found in cruciferous vegetables, by heterocyclic amines in cooked meats, and by PAHs found in grilled meat. CYP1A2 is also inhibited by the compound naringenin, which is found in grapefruit [1]. Therefore, metabolism of heterocyclic amines by the enzyme encoded by the CYP1A2 gene may depend not only on the genetic variant of CYP1A2 that is carried by an individual, but also on the individual’s intake of these other food components that affect CYP1A2 expression [13]. Similarly, consumption of a diet that is low in fat and glycemic load was shown to be associated with alterations in gene expression in the prostate epithelium in humans [6].

- Glutathione S-transferases (GSTs)

The GSTs are a major family of cytosolic enzymes that catalyze the conjugation of reduced glutathione to a large number of electrophilic compounds formed by cytochrome P450 enzymes. Because electrophiles can bind to DNA, forming adducts and potentially DNA mutations, GSTs play a critical role in protecting cells against the cytotoxic and mutagenic effects of these reactive compounds. Thus, it has been hypothesized that GST induction results in an overall decreased cancer susceptibility and that, conversely, impaired detoxification by GST will confer increased susceptibility to disease. The GSTs are divided into four major classes alpha (GSTA), pi (GSTP), mu (GSTM), and theta (GSTT), based on their physicochemical and immunologic properties, and, within each class, several isozymes exist. GSTs have been found in all human tissues studied, but with striking differences in isozyme distribution in different tissues androgens. For example, of the total hepatic GST protein, 80% is GSTA, 10–20% is GSTM, and < 5% is GSTP. In the lung, GSTP is the predominant class (85%), whereas GSTM and GSTA isozymes constitute only 7–8% each.

- Methylenetetrahydrofolate reductase (MTHFR)

MTHFR is an example of a nutrient‐gene interaction that may affect the risk of development of cancer and common chronic diseases. MTHFR is an enzyme coded for by the MTHFR gene. It plays an important role in 1‐carbon metabolism by producing 5‐methyltetrahydrofolate, a coenzyme that serves as the methyl donor needed for the conversion of homocysteine to methionine. In an allelic variation that occurs in some people (an example of small single-nucleotide polymorphism; SNP), a base pair substitution occurs at 677 of the gene that encodes methylene-tetrahydrofolate reductase (MTHFR). This results in the variant allele in which cytosine is replaced with thymine. This polymorphism is denoted as MTHFR 677C → T. This allelic variation (i.e., MTHFR 677 TT) produces a change in the enzyme that reduces its function. A recent study supports the hypothesis that individuals with the TT genotype may have higher folate requirements, and this genetic variation may modulate the risk for vascular and neoplastic diseases and neural tube defects (NTD). The risk for developing a certain disease according to an individual’s genome is influenced by many factors such as the disease in question, the individual’s diet, ethnic and geographic elements, and many others. For instance, in a Physicians’ Health Study, men with the MTHFR 677 TT genotype and adequate folate level had a 55% lower risk for colorectal cancer compared with men with either of the other 2 genotype combinations (i.e., CC or CT). This protective effect was lost when folate status was impaired. However, compared with the CC genotype, the TT genotype has been associated with a higher risk for NTDs, which may be exacerbated by low folate levels. Because SNPs are more common than single gene disorders, identifying those SNPs associated with diet‐related diseases could lead to targeted nutrition interventions according to genotype [14].

### Nutrigenomics relation to disease susceptibility

- Nutrigenomics and obesity

Genes affecting weight regulation are grouped in a number of ways. The first grouping depends on genetically regulated processes and mechanisms that contribute to body weight homeostasis. This includes physical activity, appetite, adipocyte differentiation, insulin signaling, mitochondrial functions, lipid turnover, thermogenesis, and energy efficiency.

**Fig. 2:**
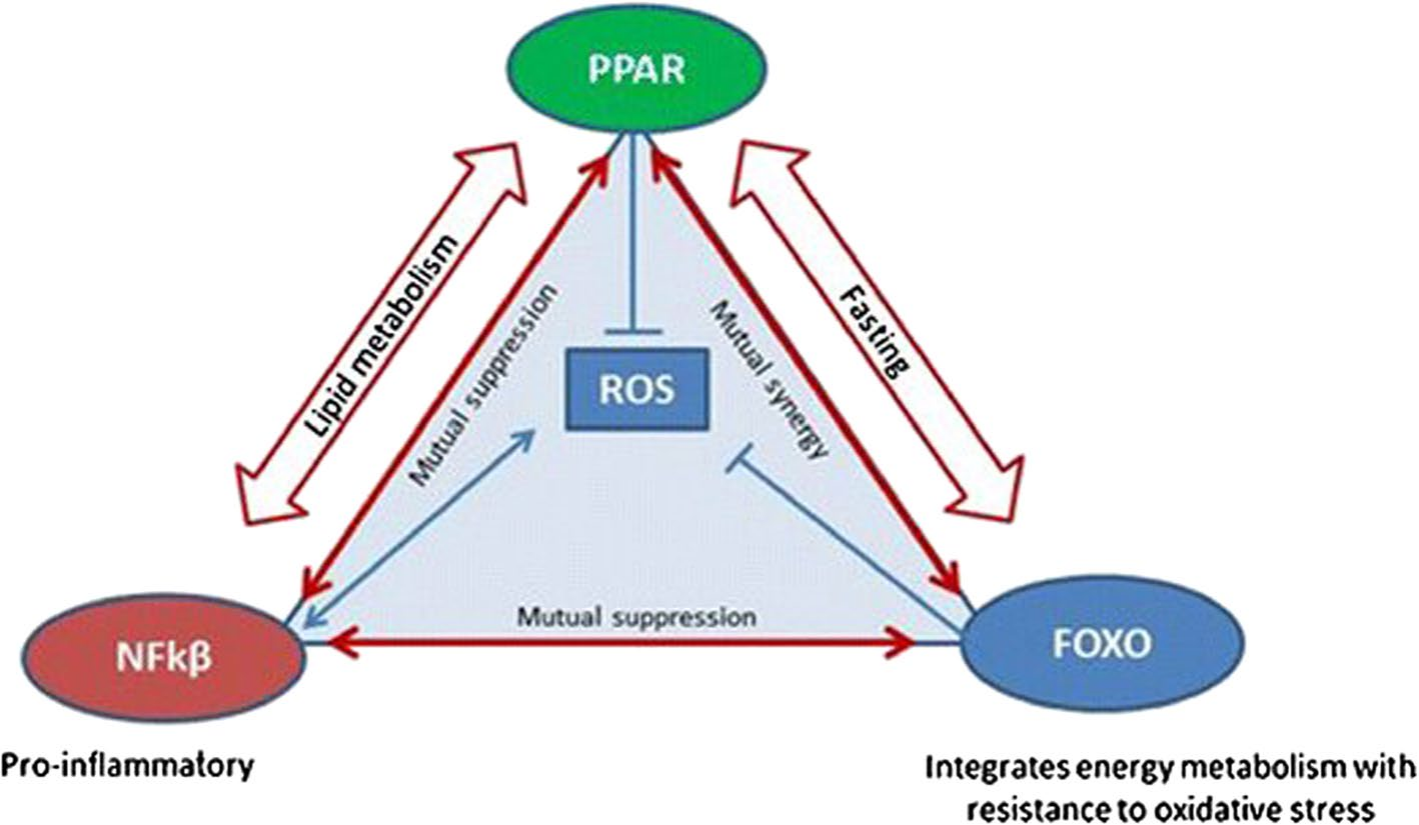
Transcriptional triad of survival [15]

Then, genes are secondly grouped according to how they regulate metabolic functions (Fig. 2), in which there are polymorphisms that have been related to genetically mediated differences to dietary weight loss interventions, including the following:

- ➢ Regulating energy intake (e.g., MC3R, MC4R, POMC, LEP, LEPR, FTO)
- ➢ Lipid metabolism and adipogenesis (e.g., PLIN1, APOA5, LIPC, FABP2)
- ➢ Thermogenesis (e.g., ADBRs, UCPs)
- ➢ Adipocytokine synthesis (e.g., ADIPOQ, IL6)
- ➢ Transcription factors (e.g., PPARG, TCF7L2, CLOCK)
- ➢ Nutrigenomics and type II diabetes mellitus

The hormone insulin, which is secreted from pancreatic β-cells, is an important controller of both glucose and fat metabolism. Impaired regulation of its secretion is observed in both obesity and type II diabetes mellitus. Diets high in sugar and saturated fatty acids elicit a condition termed “glucolipotoxicity” [16] that negatively impacts the ability of the β-cell to adequately secrete insulin, resulting in hyperglycemia and hyperlipidemia. Many of the effects of sugars and fats are mediated through transcriptional regulation of β-cell gene expression. Moreover, as the fat mass of obese individuals increases, the concentration of inflammatory mediators produced by adipocytes rises. These mediators, termed adipokines, may also influence insulin secretion directly and indirectly via genomic changes. Whether many of the adipokines are influenced by dietary factors is not well known. Improving our understanding of how nutrigenomics modifies both adipokine secretion and β-cell function may facilitate prevention or amelioration of diet-related chronic diseases, such as type II diabetes mellitus.

- Nutrigenomics and cardiovascular system (CVS) diseases

Hypertension is associated with heart failure, renal disease, stroke, and cardiovascular death and is highly modified by lifestyle changes [17]. The angiotensin-converting enzyme (ACE) is the key enzyme in controlling blood pressure, and polymorphisms in ACE, the gene encoding this enzyme, have been implicated in the risk for hypertension and CVS diseases. In the Nutrigenomic Analysis in Twins (NUGAT) study, GG genotype of rs4343 of ACE was associated with increased risk for hypertension and CVS diseases in individuals on high saturated fat diet [18]. The proposed mechanisms seem to involve high-fat in activating RAS in adipocytes through toll-like 4 receptors and nuclear factor kappa B (NFkB) signaling [19]. The association of salt with hypertension is well-known, and there have been some efforts to elucidate the genetic contribution to salt-sensitive blood pressure (SSBP) changes. In a recent study, variants in estrogen receptor (ESR2) were implicated in SSBP. Estrogen-replete women with ESR2 rs10144225 minor allele were more salt-sensitive than men or postmenopausal women [20]. The Genetic Epidemiology Network of Salt-sensitivity (GenSalt) study was designed to understand the basis of salt-sensitivity in ~ 2000 individuals administered a 7-day low-sodium diet followed by a 7-day high-sodium diet. This study found SSBP to be associated with rare variants in three genes of a group of renal epithelial sodium channels (ENaC).

### Relation between blood type and diet

There is a new hypothesis called “blood-type” diet. It says that if everyone eats according to their blood type, people will be healthier and live longer. It postulates that type O needs high protein diet with lots of meat, vegetables and fish. Type A should choose fruits, vegetables, tofu, and avoid meat. Type B can take variety of diet including meat, fruits, seafood and grains. Type AB should eat dairy, tofu, lamb, fish and grains. Studies made on this hypothesis were inconclusive, but one study on ABO genotype and cardio-metabolic risk factor revealed that adherence to blood type diet had favorable effects on some cardio-metabolic risk factors as lowering BMI, waist circumference, blood pressure, serum cholesterol, triglycerides, and insulin with variations between blood types. However, it was also found that there is no significant association between these changes and blood type and these results does not support the “blood-type” diet hypothesis [21].

### Nutrigenomics and cancer

- Gastro-intestinal tract (GIT) cancers

### Esophageal cancer

In study done in China on the etiology and prevention of ESCC, esophageal squamous-cell carcinoma which comprises 60–70% of all cases of esophageal cancer worldwide, diet was one of the most important risk factors besides family history and infections [22]. Consuming hot beverages and pickled food, part of traditional Asian food, significantly increased the risk among the Asian population. Red and processed meat were also associated with a 57% and 55% increased esophageal squamous cell carcinoma (ESCC) risk, respectively, compared to those who consumed low amount of meat. Fruits and vegetables intake was associated with a 30% decreased risk. The study also noted that lack of some micronutrients may be associated with increased risk. For, intake of β-carotene, vitamin E, and selenium showed a reduction in ESCC mortality by 17%. Additionally, moderate supplementation of riboflavin (a micronutrient found in eggs, green vegetables, and organ meat such as kidney and liver) will decrease risk, prevent recurrence of ESCC, and improve the prognosis of ESCC patient [23].

### Stomach cancer

It is heavily associated with *Helicobacter pylori (H. pylori*) infection and smoking that stomach cancer was also linked to certain nutrients in many studies. In a recent systemic review and meta-analysis published recently [24], stomach cancer was strongly attributed to high intake of salty and pickled foods. Alcohol drinking, especially for long periods, was distinctively connected even after the subjects quitted, showing an increased risk by 19%. Something could be explained by the permanent damage caused by alcohol on the stomach lining and liver after long periods of heavy drinking. Coffee and green tea showed no significant association. Fruits and vegetables decreased risk of stomach cancer by 48% and 62% respectively.

### Colorectal cancer

Colorectal cancer (CRC) is the third most common cause of cancer-related deaths which contributes to a significant public health problem worldwide with 1.8 million new cases and almost 861,000 deaths in 2018 according to the World Health Organization. It exhibits 7.4% of all diagnosed cancer cases in the region of the Middle East and North Africa [25]. Of all cancer-related deaths, 25–30% can be attributed to tobacco, 30–35% are linked to diet, and 15–20% are due to infections [26]. Specifically for colon cancer, intestinal inflammatory diseases (such as Crohn’s disease and ulcerative colitis) and obesity are additional risk factors. One of the most related dietary factors to colorectal cancer is the intake of red and processed meat. There are multiple proposed explanations for this phenomenon. First, red meat contains high levels of heme, iron-porphyrin pigment. Heme is poorly absorbed by the small intestine which makes it accumulate in the colon. Chronic accumulation of heme induces colonic injury, resulting in hyper-proliferation and hyperplasia, which may lead to the development of colorectal cancer. Eventually, heme may lead to the development of colorectal cancer by altering the microbial composition of the colonic epithelial cells and thus leading to their malignant transformation [27]. The second potentially harmful compound in red meat is heterocyclic amines, which are produced when red meat is cooked at high temperatures. When these heterocyclic compounds enter the cell, they get metabolized into toxic compounds that interact with DNA. This results in DNA mutations in some oncogenic genes such as adenomatous polyposis coli (Apc), β-catenin, and K-Ras [28]. The third class of compounds linked to colorectal cancer is N-nitroso compounds (NOCs), which are also found in processed meat. Similar to heme and heterocyclic amines, these compounds interact with the DNA to induce mutations in key genes. Recent epidemiological studies linked NOCs to increased risk of colorectal cancer [29, 30]. Also, diet and obesity are important risk factors for CRC; excessive body fat leads to increased insulin resistance hyperinsulinemia that leads to increased insulin growth factor 1 (IGF-1) and causes low grade chronic inflammation. Also insulin increases ovarian androgen synthesis and growth hormone receptor expression and inhibits liver synthesis of binding proteins leading to greater bioavailability of IGF-1 [31, 32]. IGF-1 activates phosphatidylinositol-3-kinase (PI3K)/Akt signaling pathway that increases cell proliferation [33]. Also, excessive body fat increases levels of leptin, which regulate PI3K/Akt signaling and reduce adiponectin. Also, the low-grade chronic inflammation makes a good environment for carcinogenesis. It is believed to be mediated by TNF-a, CRP, and IL-6. Adiponectin reduces TNF-a, while insulin increases IL-6 secretion which leads to abnormal cell proliferation and carcinogenesis [33].

- Breast cancer

Breast cancer (BC) is the 2nd most prevalent and lethal malignancy worldwide. For long, mainly alcohol and smoking have been recognized as major lifestyle risk factors in the development of breast cancer. However, recent studies showed that different dietary components may affect the development and prognosis of breast cancer [34]. For example, there is a positive correlation between meat intake (processed and red meat) and risk of developing breast cancer Similarly, women consuming diets high in saturated fats and cholesterol had an increased risk too. On the contrary, consumption of fish was associated with a significant reduction of breast cancer risk. A cohort study found that marine n-3 polyunsaturated fatty acids resulted in 14% reduction in breast cancer risk [35]. Also, omega-3/omega-6 lipids correlated to a decreased risk [36]. Concerning dairy intake, there is evidence that moderate and high intake are associated with a decreased risk. However, only yogurt and low-fat dairy had protective effects, unlike other dairy products [37]. Concerning fruits and vegetables, some studies showed no correlation between consumption and risk, while others demonstrated that apple and citrus fruits may exert a protective effect [38]. Regarding tea and coffee, there is no clear effect. However, alcohol is associated with higher risk and poor prognosis [39]. Mediterranean and vegetarian diets also showed no clear effects, having a protective value in some studies but not in others. However, a meta-analysis reported a protective role of consumption of cruciferous vegetables [40]. Additionally, intake of soy, an important source of phytoestrogens and isoflavones, was associated with decreased risk [41].

- Prostate cancer

Epidemiology studies of prostate cancer have strongly implicated the diet as a major modulator of prostate cancer risk [42]. The incidence of prostate cancer varies across different geographic regions. For example, it is high in USA and Europe, but low in Asia. However, when Japanese immigrants in the USA were examined, they showed high-risk criteria for prostate cancer [43, 44]. This could be explained by the fact that when those immigrants adopted certain dietary habits, they became at risk. The most consistent dietary factor associated with prostate cancer is the intake animal fats, red meat, and dairy products [45]. Also, in a meta-analysis study that was done recently, intake of calcium was also associated with increased risk [46]. On the other hand, like breast cancer, soy intake was correlated with decreased risk [47]. Some specific food components affecting development of cancers are described in Table 1.

**Table 1:** Specific food components affecting development of cancers

Food factors increasing cancer risk	Food factors decreasing cancer risk
Charcoal-broiled meat: stomach, colon, and rectal cancers [48]	Vitamin A and beta-carotene: lung, stomach, colon, prostate, and cervix cancers [49]
Red meat: colorectal cancer [48]	Vitamin C: esophagus, stomach, rectum, pancreas, breast, cervix, and lung cancers [49]
Nitrites: stomach cancer [50]	Vitamin E: colon and prostate cancers [49]
Salt: stomach and throat cancer [48]	Selenium: lung, prostate and colon cancers [49]
Saturated fat and trans-fatty acids: lung, colon, rectum, breast, and endometrium cancers [51]	N-acetyl-cysteine (NAC): blocks the metastatic potential of several cancer cell lines through inhibition of enzymes that stimulate tumor vascularization [52]
Alcohol: liver, breast, colon, rectum, oral, and esophageal cancers [53]	Cruciferous vegetables: colorectal cancer [49]
Sweeteners: saccharin, cyclamate Aspartame: lymphoma, leukemia, and bladder tumors [54]	Resveratrol: breast, blood and lung cancers [55]
Food additives: potassium bromate, fluoride Food dyes: osteosarcoma, bladder, kidney, and thyroid tumors [56]	Curcumin: leukemia, lymphoma, stomach, gastrointestinal, sarcoma, breast, and head and neck cancers [57]
Glyphosate: non-Hodgkin lymphoma and pancreatic cancers [58]	Lycopenes: prostate cancer [59]

### Cancer prevention

The use of nutrigenomics in cancer prevention is increasing widely. Food and dietary components can change the track of cancer pathogenesis and prognosis. They exert their effect through different mechanisms in carcinogen metabolism, DNA repair, cell proliferation/apoptosis, inflammation, oxidant/antioxidant balance, and angiogenesis [60]. Also, some key micronutrients as (vitamin A, vitamin C, vitamin D, and selenium) can have anti-carcinogenic properties and can be used in cancer therapy [61]. DNA repair is very important to keep the genome stable and prevent mutations that can lead to cancer. Dietary food components were found to play a role in maintaining DNA repair, and their deficiency leads to disruption in the repair leading to damage and mutations of DNA. For instance, flavonoids, vitamins E and C, and isothiocyanates stimulate repair of DNA damage from ROS (reactive oxygen species) [62]. Adding cooked carrots in food increases the repair of 8-oxodG (a marker of oxidative stress-derived DNA damage) in white blood cells [63]. Another mechanism of prevention is inducing an arrest of the cell cycle progression [64]. Some dietary compounds can attack the cell cycle at any stage resulting in its arrest. These compounds are phenolic compounds such as genistein, epigallocatechin-3-gallate and also isothiocyanates [65, 66]. Dietary compounds also induce apoptosis in the cells through a mitochondrial pathway which leads to release of cytochrome C from mitochondria and activation of caspase-3, caspase-6, and caspase-7 finally ending in apoptosis [66]. In chronic inflammations, cell damage can lead to DNA mutations and cause malignancy [67]. The changes that lead to damage include DNA damage, disruption of DNA repair pathways, cellular proliferation, inhibition of apoptosis, and promotion of angiogenesis and invasion. Some dietary food components are found to target these changes and prevent damage. Another mechanism where dietary components can inhibit carcinogenesis is by preventing of angiogenesis. Polyunsaturated fatty acids and polyphenols, such as epigallocatechin-3-gal-late, resveratrol, curcumin, and genistein, are compounds that have a role in angiogenesis prevention [68–70].

### Role of some key micronutrients in cancer prevention

#### Vitamin C

Vitamin C or ascorbic acid is a vitamin found in various foods and sold as a dietary supplement. Vitamin C has dose-dependent anticarcinogenic properties [71]. In specific cancers, such as melanoma, if it is administered in high doses, it induces apoptosis, but in low doses, it promotes cell proliferation [72, 73]. However, high doses can have side effects due to the pro-oxidant action when accumulated in cells. Also, it increases the sensitivity to chemotherapy, thus lowering the doses of chemotherapeutic drugs needed to obtain certain results [74]. Another anti-carcinogenic mechanism is by acting as antioxidant. High doses of vitamin C produce high amounts of hydrogen peroxide that accumulate in cells and can lead to death of cancer cells through induction of apoptosis [75]. Besides its role in cancer prevention, studies have shown that it also has an impact on cancer-related mortality rates in breast cancer [76].

#### Vitamin A

Vitamin A is a group of unsaturated nutritional organic compounds that include retinol, retinal, and several provitamin A carotenoids. It works as an antioxidant that protect against oxidative stress preventing DNA damage. It also regulates methylation resulting in controlling cell growth. It was found that it decreases some of the side effects of chemotherapy as mucositis [77]. It is also able to reduce head, neck, and lung carcinogenesis [78] and inhibit premalignant lesions by regulation of genes involved in cell growth and differentiation. Retinoids and lycopene play an important role in oral cancer prevention and treating oral leukoplakia [79]. Plus, when retinoids are combined with bexarotene, they reduce chemical induction of oral carcinogenesis by ROS prevention [80]. It is also proved to have preventive roles in lung and pancreatic cancers.

#### Vitamin D

Vitamin D was found to have protective properties in oral, head and neck, breast, ovarian, prostate, and colon cancers [81, 82].

It works on both the genomic level, via the vitamin D receptor (VDR), and on the cellular level. Vitamin D has a role in cellular immunity protecting against pathogens. It protects against pancreatic cancer by inhibiting proliferation and angiogenesis [83]. It increases radio sensitization in breast cancer and promote apoptosis in oral cancer [84].

#### Folic acid

It is controversial if folic acid is pro or anticarcinogenic. Folic acid has a role in DNA, RNA, and protein methylation as well as keeping DNA integrity [85]. MTHFR is implicated in the metabolism of folic acid catalyzing the synthesis of 5-methyl tetrahydrofolate, and any change in this methylation process can lead to cell aging and carcinogenesis [86]. Folic acid also was found to have preventing effects in colorectal cancer and to improve results of chemotherapy in lung cancer [87, 88].

#### Vitamins and viral infections

Vitamins are have shown a great effect in improving the immunity and enhancing the defense mechanism of the body against viral infections, and this role sparkled in the time of COVID-19 when supplementation with vitamins was essential in every prescription to a corona virus patient. The most prominent one was vitamin D. Vitamins D decreases the risk of viral infection and mortality through three pathways: physical barrier, cellular immunity, and humoral immunity. It reduces the release of the cytokines, thus improving the cellular immunity, and regulates the humoral immunity through inhibiting the release of T helper cells and stimulating the regulator T cells induction [89]. Another one is vitamin A “the anti-infection vitamin” which has a role in phagocytic and oxidative mechanisms and regulating natural killer (NK) cells, IL-2, and the pro-inflammatory TNF-α [90]. Vitamin E, vitamin C, vitamin B1, and vitamin B6 also proved to have beneficial effects in treatment of corona virus [91].

### New directions in the application of micronutrients against cancer: synergy approach

Recently, there is a new direction to use combined micronutrients to get synergistic action with better results in defending against cancer, through activating different mechanisms simultaneously [92]. These combinations were used in studies of colon, breast, skin, kidney, and liver cancers and showed inhibition of cell proliferation, suppression of tumor growth and invasion, inhibition of angiogenesis and metastasis [93, 94], and inducing apoptosis in other types of cancers [95, 96]. It was proven that their synergistic action was superior to the individual action of these components [97].

## Conclusions

The association between food constituents and cancer is getting clearer. The strong relation between diet and cancer should direct us to ask important questions when examining people at risk of different diseases. We should think about the following: What do these people eat? Is their diet correlated to the risk and development of cancer? And if yes, can this correlation be generalized or does it differ according to certain genotypic, ethnic, and/or geographical variabilities? And, finally, can a certain diet enhancement affect their prognosis and treatment? These and so many other question should be noted in cancer research in the future.

This will also help optimize diet for each individual, taking into account the disparity in metabolic requirements and gene expression each person holds. Eventually, it will become more about personalized nutrition and less about one-size-fits-all “good” diets, improving diet qualitatively and quantitatively.

Food components affect carcinogenesis almost in all its stages from initiation of the disease by gene mutation to controlling the progress and even prevention of cancer, playing an important role in cancer therapy. Some food constituents are carcinogenic, while others are protective through different mechanisms which need to be thoroughly investigated with new technologies in oncology to be applied in cancer therapy. Also, we discussed how the protective potential depends on doses (as some nutrients are preventive in high doses and may be harmful in low doses as mentioned above), time of administration, and combination with other compounds (synergistic action). All these factors, if properly studied and applied, may take cancer therapy another level.

## Data Availability

The datasets used and/or analyzed during the current study are available from the corresponding author on reasonable request.

## Abbreviations

ACE: Angiotensin-converting enzyme
ADBRs: Adrenergic receptors
ADIPOQ: Adiponectin, C1Q and collagen domain containing
Apc: Adenomatous polyposis coli
APOA5: Apolipoprotein A5
BC: Breast cancer
BMI: Body mass index
CLOCK: Clock circadian regulator
CRC: Colorectal cancer
CVS: Cardiovascular system
CYP: Cytochromes P450
D.M: Diabetes mellitus
DNA: Deoxyribonucleic acid
ENaC: Epithelial sodium channels
ESCC: Esophageal squamous cell carcinoma
ESR2: Estrogen receptor
FABP2: Fatty acid binding protein 2
FDA: Food and Drug Administration
FTO: Fat mass and obesity-associated gene
GenSalt: Genetic Epidemiology Network of Salt-sensitivity
GIT: Gastro-intestinal tract
GIT: Gastro-intestinal tract
GSTs: Glutathione S-transferases
*H. pylori*: *Helicobacter* Pylori
IGF-1: Insulin growth factor 1
IL6: Interleukin-6
LEP: Leptin
LEPR: Leptin receptor
LIPC: Lipase C, hepatic type
MC3R: Melanocortin 3 receptor
MC4R: Melanocortin 4 receptor
MTHFR: Methylenetetrahydrofolate reductase
NAC: N-acetyl-cysteine
NFkB: Nuclear factor kappa B
NK: Natural killer
NOCs: N-nitroso compounds
NTD: Neural tube defects
NUGAT: Nutrigenomic analysis in twins
PI3K: Phosphatidylinositol-3-kinase
PLIN1: Perilipin 1
POMC: Proopiomelanocortin
PPARG: Peroxisome proliferator activated receptor gamma
ROS: Reactive oxygen species
SNP: Single nucleotide polymorphism
SSBP: Salt-sensitive blood pressure
TCF7L2: Transcription factor 7 like 2
TNF-a: Tumor necrosis factor alpha
UCPs: Uncoupling proteins
VDR: Vitamin D receptor
